# Factors triggering queen executions in the Argentine ant

**DOI:** 10.1038/s41598-019-46972-5

**Published:** 2019-07-18

**Authors:** Sílvia Abril, Crisanto Gómez

**Affiliations:** Department of Environmental Sciences, University of Girona, M. Aurèlia Campmany, 69, 17003 Girona, Spain

**Keywords:** Entomology, Animal behaviour

## Abstract

Competition among queens in polygynous societies may result in queen executions or conflicts over personal reproduction. Understanding the factors that mediate the executions of ant queens should provide insight into how queen numbers are regulated in polygynous insect societies. The Argentine ant is a widespread invasive species that displays secondary polygyny, and workers execute 90% of their nestmate queens each spring. In this study, we investigated: (1) whether ambient temperature, queen number, and protein deprivation have an effect on queen executions and (2) whether workers select the queens slated for execution based on their cuticular hydrocarbon (CHC) profiles. We found that the percentage of queens executed was positively correlated with temperature and queen number but that protein deprivation did not play a role. As for queen fate, the levels of some CHCs were higher in surviving queens. One of these CHCs is associated with queen productivity (i.e egg-laying rate and ovarian index) suggesting that workers execute the least productive queens. Our findings suggest that chemical cues related to fertility signaling may mediate queen executions in Argentine ants.

## Introduction

Among social insects, ants stand out as a group in which polygyny (i.e., the presence of several queens in the same colony) may be the predominant social structure^1^. Polygyny in ants can be primary or secondary^2,3^. Primary polygyny, the less frequent of the two, occurs when multiple queens without workers collaboratively found a new colony (pleometrosis), which means that the foundresses co-exist during the colony’s initial stages. Primary polygyny can also occur when multiple queens accompanied by workers found a new colony after budding from larger colonies^2^. Secondary polygyny is more common and occurs when monogynous colonies—established either via claustral colony founding or via budding from larger polygynous colonies^2,3^—become polygynous after gyne acceptance and/or after colony fusion^4–7^. Executions may then be used to reduce queen numbers and thus maximise egg production^8^.

Polygyny can lead to reproductive competition among queens, which can result in queen executions or conflicts over personal reproduction. Queen-queen competition occurs in both primary and secondary polygyny but manifests itself differently in the two systems. In primary polygyny, there are fights among queens or between queens and workers after which a single queen remains^9–12^; all the others are executed. In secondary polygyny, in contrast, there is a decrease in queen fecundity as queen numbers increase^13–17^. Among ants, this negative effect of queen number on the fecundity of individual queens has frequently been observed in several species^18^. According to Vargo^17^, there are two likely mechanisms by which increased queen numbers could diminish individual fecundity. First, if there are more queens sharing the same given amount of resources, each queen might receive less food and thus create fewer eggs. Second, there might be pheromone-mediated reproductive competition among queens.

The Argentine ant, (*Linepithema humile*, Mayr), an invasive species found across the globe, displays secondary polygyny and carries out a dramatic number of queen executions. Every spring, workers execute about 90% of their nestmate queens, resulting in a 7% loss in the colony’s total biomass^19,20^. Previous studies examined if physiological factors such as mass, egg-laying rate, and the quantity of sperm stored differed between surviving and executed queens, but no relationship was found^20^. Reuter *et al*.^21^ also explored whether relatedness played a role in queen executions; they found that workers were, on average, equally related to surviving and executed queens. Queen executions therefore do not seem to constitute a mechanism for increasing the inclusive fitness of workers^21^. That said, Inoue *et al*.^22^ showed that genetic diversity in Argentine ant workers significantly decreased from May to September and suggested that queen executions are a contributing factor.

In the Argentine ant, as in other ant species, queen pheromones can inhibit gyne development^23–26^. The spring execution of queens seems to respond to the workers’ need to lower queen pheromone levels so that larvae can undergo sexualisation^27^. Consequently, the concentration of queen pheromones in colonies is thought to play a key role in regulating queen numbers, especially in highly polygynous species such the Argentine ant. Deslippe and Guo (cited in^28^) found that, in fire ants, the mortality rate of queens was positively correlated with queen number, which could suggest that workers execute supernumerary queens to regulate the level of queen pheromones circulating in the colony.

In the Argentine ant, colony queen number is influenced by seasonal nest displacement^29^ and by invasion history^30^. In the latter case, it is higher along the invasion front than in fully invaded zones from which the native ant community has almost entirely disappeared^30^. These differences seem to stem from a strategic response: the species is seeking to quickly colonise nesting sites along the invasion front, where resources are more limited due to competition with the native ant community. Consequently, the question arises: what are the patterns of spring queen executions along the invasion front versus behind the invasion front? For example, are more queens executed in one location versus the other, or are numbers the same in both places?

Protein availability may also be involved in regulating queen numbers, especially during the execution period. In the Argentine ant, queens are fed mostly protein-rich foods^31^. It has been estimated that a queen receives 275 times more protein than does a worker and twice as much as a worker larva^31^. Protein-rich food is essential for the optimal growth and development of larvae and for the production of eggs by queens^31^. It is known that Argentine ant queens inhibit the production of males by appropriating food, as male larvae will fail to pupate if they are protein deprived^32^. Given this information, it seems possible that the low worker/larva ratios in nests in the spring could trigger queen executions: the presence of fewer queens would increase the amount of protein-rich food available for sexualised larvae. To our knowledge, the role of protein availability in regulating queen executions is unknown.

Temperature might also influence queen executions. First, this factor can affect gyne production. Overwintering has a stimulatory effect on Argentine ant larvae. More specifically, it increases their tendency to sexualise, and it affects the rearing behaviour of workers such that they rear bipotent larvae as gynes^27^. Second, in the Argentine ant, temperature regulates the foraging activity of workers^33^; the oviposition rate of queens^15^; and the developmental time of brood^34^. Third, temperature can also induce changes in pheromone biosynthesis, emission, dispersal and perception^35^ and, therefore, affect the mechanisms used by individuals to recognise each other. In the Argentine ant, temperature, acting through shifts in pheromone mediated communication, could potentially affect the aggressiveness displayed by workers towards their nestmate queens. Once again, however, nothing is known about the effect of temperature on queen executions.

In addition to better understanding the factors that trigger queen executions in the Argentine ant, it is also important to clarify how a queen’s fate is determined. A recent study has suggested that Argentine ant workers may cull queens based on their cuticular hydrocarbons (CHCs)^36^. However, the CHC data for executed and surviving queens were obtained by taking samples of queens before and after the execution period in the field. Directly analysing the CHC profiles of executed queens could provide more information about how workers decide a queen’s fate.

The aim of this study was to improve our understanding of the mechanisms that underlie social organisation in the Argentine ant, a globally invasive species that displays secondary polygyny. More specifically, we were interested in clarifying the factors that help regulate queen abundance and the basis for queen fate. We thus investigated:

1. The potential effects of ambient temperature, queen number, and protein deprivation on queen executions. We hypothesized that an increase in ambient temperature and queen number might trigger a higher level of queen executions by affecting pheromone biosynthesis, release, diffusion, and perception, which may be greater at higher ambient temperatures and queen numbers. We also hypothesized that protein deprivation in colonies with low worker/larva ratios could trigger queen executions so as to promote larval development.
2. The potential effect of queens’ CHC profiles on the selection of the queens to be executed. Based on a previous study that suggested that Argentine ant workers may cull queens based on their CHCs, we hypothesized that the CHC profiles of executed queens would be different from those of surviving queens and that these differences would determine queen fate.

## Results

### Effect of temperature and queen number on queen executions

**Figure 1:**
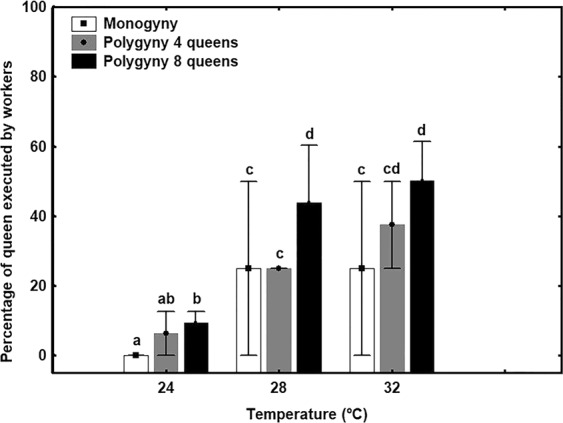
Percentage of queens (mean ± SE) executed by workers according to ambient temperature (24 °C, 28 °C, and 32 °C) and queen number. White bar: monogynous nests; grey bar: polygynous nests with four queens; black bar: polygynous nests with eight queens. The different letters denote significant differences (p < 0.05).

A significantly higher percentage of queens were killed in the colonies kept at 28 °C and 32 °C than in the ones kept at 24 °C (GLMM: t = 2.47, p < 0.01; results of paired t-tests: 28 °C and 32 °C vs. 24 °C—p < 0.05) (Fig. 1). Also, a greater percentage of queens were killed in polygynous nests with eight queens than in monogynous nests (GLMM: t = 1.96, p < 0.05; results of paired t-tests: monogynous vs. polygynous with eight queens—p < 0.05) (Fig. 1).

**Figure 2:**
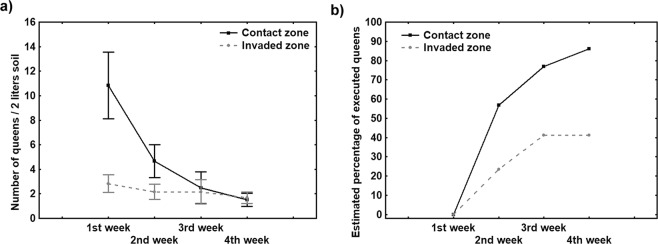
Weekly changes (mean ± SE) in the number of queens (**a**) and the estimated percentage of queens executed (**b**) in May in the contact zone and the invaded zone.

In the field, the number of queens per two litres of nest soil in May was markedly different between the contact zone and the invaded zone (mean queen number in early May: contact zone = 10.8 ± 7, invaded zone = 2.8 ± 1.8; GLMM: t = −3.40, p < 0.01). It also differed significantly over time—it grew smaller as the weeks went by (GLMM: t = −3.50, p < 0.01). In addition, there was a significant interaction between these two factors (area and week). This result indicated that the decrease in the number of queens differed between the two areas across time and that, more specifically, it was more dramatic in the contact zone (GLMM: t-value = 2.27; p < 0.05) (Fig. 2a).

The slopes of the regression lines for the mean number of queens present per week in May were markedly different between the two areas (contact zone: y = −3.17x + 12.42; invaded zone: y = −0.4x + 3.08), which underscored that the decline was more dramatic in the contact zone. Furthermore, although the two areas displayed differences in the number of queens present in early May, these differences had disappeared by the end of May (mean ± SD number of queens in two litres of nest soil: contact zone = 1.5 ± 1.38; invaded zone = 1.6 ± 1.21). Given that the decrease in queen numbers was most likely due to queen executions, we estimated that 86.15% of queens were eliminated in the contact zone, as compared to 43.53% of queens in the invaded zone (Fig. 2b).

### Effect of protein deprivation on queen executions

Protein deprivation did not play a significant role in queen executions because there was no difference in the percentage of queens executed by colonies given a protein-rich diet versus by colonies given a protein-free diet (GLMM: t = −0.012, p = 0.989). Interestingly, larvae in the latter colonies did not die. Instead, they pupated earlier than larvae from the former colonies. Consequently, the workers that emerged from these pupae were smaller than average (pers. obs.).

### Relationship between CHC profiles and queen fate

**Figure 3:**
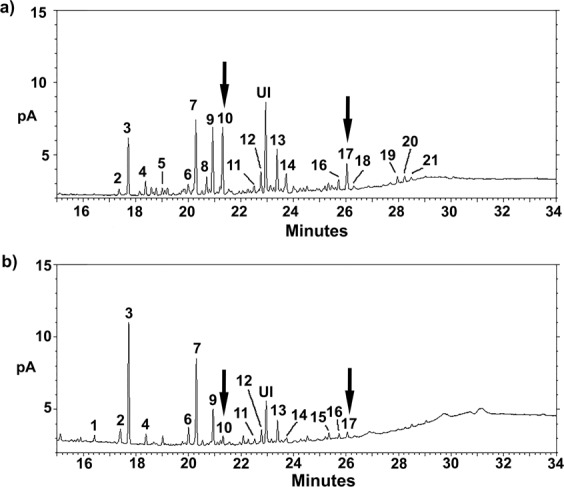
Example CHC profiles for a surviving queen (**a**) and for an executed queen (**b**). Compound names are provided in Table 1. UI: unidentified non-hydrocarbon compound. Arrows indicate the compounds 5,11-diMeC_29_ and 5,11-diMeC_33_, respectively.

**Figure 4:**
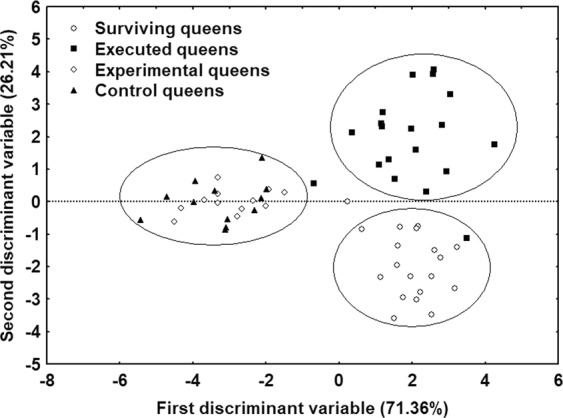
Discriminant analysis conducted using the relative quantities of CHCs in the profiles of (1) surviving and executed queens and (2) experimental versus control queens from the supporting experiment controlling for the effect of death on the profiles.

**Figure 5:**
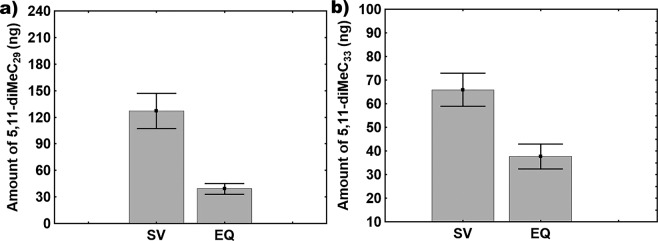
Amount of 5,11-diMeC_29_ (**a**) and 5,11-diMeC_33_ (**b**) in the CHC profiles of surviving and executed queens (mean ± SE). SV: surviving queens; EQ: executed queens.

There were significant differences in the CHC profiles of executed versus surviving queens (Figs 3 and 4). Two discriminant functions explained most of the variance (global model: Wilk’s λ = 0.031, F = 5.9, p < 0.0001; 85.45% correct classification; first function: R = 0.93, Wilk’s λ = 0.031, χ^2^_48_ = 176.50, p < 0.0001; second function: R = 0.841, Wilk’s λ = 0.238, χ^2^_30_ = 73.10, p < 0.0001). The second discriminant function fully differentiated between them: executed queens were clustered at one end of the spectrum and surviving queens at the other (Fig. 4). The main CHCs responsible for these differences were two di-methyl alkanes (5,11-diMeC_29_ and 5,11-diMeC_33_), a single alkene (C_31:1_), and one mono-methyl alkane (5-MeC_31_). Given that the relative quantities of these four compounds differed between executed and surviving queens, we examined their absolute quantities. Overall, surviving queens had higher quantities of 5,11-diMeC_29_ and 5,11-diMeC_33_ than executed queens (5,11-diMeC_29_: GLMM: t = −4.09, p < 0.005; 5,11-diMeC_33_: GLMM: t = −3.18, p < 0.01) (Fig. 5; Table 1).

**Table 1:** Relative proportions (mean ± SE) of the major cuticular hydrocarbons of surviving queens (*N* = 19) and executed queens (*N* = 19) as identified by GC-MS. Compounds are ordered by retention time. For peak numbers, refer to Fig. 3.

Peak number	Compound	Surviving queens	Executed queens
1	*n*-C_26_	0.28 ± 0.20	0.74 ± 0.10
2	xC_27:1_	1.09 ± 0.11	1.96 ± 0.35
3	*n*-C_27_	13.01 ± 0.84	14.31 ± 1.83
4	5-MeC_27_	1.89 ± 0.27	1.28 ± 0.13
5	n-C_28_	1.33 ± 0.31	1.17 ± 0.57
6	xC_29:1_	3.71 ± 0.46	2.86 ± 0.47
7	*n*-C_29_	18.06 ± 0.96	14.74 ± 1.66
8	11-MeC_29_	1.53 ± 0.18	0.62 ± 0.12
9	5-MeC_29_	7.62 ± 0.68	5.03 ± 0.65
10	5,11-diMeC_29_	4.71 ± 0.57	1.76 ± 0.42
11	xC_31:1_	1.94 ± 0.25	1.87 ± 0.24
12	*n*-C_31_	4.65 ± 0.35	4.12 ± 0.36
13	5-MeC_31_	5.13 ± 0.50	6.35 ± 1.53
14	5,11-diMeC_31_	1.69 ± 0.19	1.41 ± 0.25
15	*n*-C_33_	0.84 ± 0.16	1.32 ± 0.44
16	5-MeC_33_	1.69 ± 0.17	1.13 ± 0.11
17	5,11-diMeC_33_	2.58 ± 0.22	1.47 ± 0.34
18	*n*-C_34_ + 5,13,17-triMeC_33_	0.60 ± 0.05	1.84 ± 0.56
19	5,19-diMeC_35_	2.31 ± 0.71	2.50 ± 1.34
20	5,15 + 5,17-diMeC_35_	0.76 ± 0.08	0.44 ± 0.09
21	5,13,17 + 5,15,19-triMeC_35_	0.60 ± 0.07	1.64 ± 0.36

## Discussion

This study has revealed new information about how queen numbers are regulated in the Argentine ant, a globally invasive species that displays secondary polygyny. We found that higher queen numbers and higher temperatures resulted in a greater percentage of queen executions. These results support that queen pheromone concentration plays a key role in regulating queen numbers, and, therefore, in precipitating queen executions^27^. According to Vargo and Passera^27^, in natural populations of the Argentine ant, queen numbers are regulated by controlling the levels of inhibitory pheromone present in the colony. In this way, the development of gyne-potent larvae—which are present throughout the year—is inhibited for most of the year because more queens means more queen pheromones. In the spring, the stimulatory effect of overwintering increases the tendency of workers to rear gynes from gyne-potent larvae; it also enhances the developmental potential of larvae. The massive execution of queens has been observed in invasive populations of the Argentine ant across the globe^19,20,29^. The execution period coincides with the period of larvae sexualisation, suggesting that queens must be executed to decrease levels of the inhibitory queen pheromone and thus allow gyne production. Gynes mature and mate in the colony a few days after they emerge^37^. Consequently, queen number quickly climbs again. As the young queens begin to produce queen pheromone soon after mating^26^, its inhibitory influence prevents the appearance of new queens until the following spring. As a result, the pattern that we observed here—that queens are executed when their numbers are high—could result from workers regulating queen numbers based on the level of queen pheromones circulating in the colony. Indeed, Fletcher and Blum^4^ proposed that, in the fire ant, when levels of queen pheromones were too low or too high, workers would be spurred to bring the concentration back within the optimal range by either adopting new queens or executing supernumerary queens. It seems possible that queen pheromones could play a key role in the regulation of queen numbers in the Argentine ant as well; it could explain why, during the execution period, the decrease in queen numbers was higher in the contact zone than in the invaded zone. Queen pheromone levels seem to be the factor that determines how many queens are killed in the colony. Therefore, because nests in the contact zone have greater numbers of queens, workers are detecting higher levels of queen pheromones, and, as a result, they execute a larger number of queens. This hypothesis is consistent with our finding that both areas had the same median number of queens—approximately 1.6 queens per two litres of nest soil—at the end of the execution period. It is probable that this queen number represents the threshold at which larvae overcome the weakened inhibitory control of the few surviving queens. The larvae can thus continue their development, new gynes appear in the nest, and the executions cease.

Temperature also affects queen executions in the Argentine ant. We found that the percentage of queens executed increased as the ambient temperature rose from 24 °C to 28 °C and 30 °C. Ants, like most other insects, are ectothermic and poikilothermic, which means that body temperature changes will have a large influence on enzymatic activities such as pheromone biosynthesis or pheromone emission^35^. For example, a rise in temperature from 15 °C to 30 °C increases the emission of sex pheromones by 70% in female cabbage looper moths (*Trichoplusia ni*)^38^. Likewise, infochemical emission by ladybeetle larvae was also higher when larvae were exposed to rising temperatures^39^. To our knowledge, nothing is known about the effect of temperature on pheromone biosynthesis or emission in ants. However, based on these previous studies, it seems reasonable to hypothesise that temperature influences the emission of queen pheromones in the same way. Therefore, we posit that the increase in temperature led to an increase in queen pheromone levels, which led, in turn, to an increase in queen executions.

In contrast, protein deprivation does not seem to be an important factor in queen executions. In the Argentine ant, proteins provide amino acids that are predominately used to enhance larval growth and queen egg laying^31^. It has been shown that, in several ant species, workers harvest more protein-rich food when larvae densities are at their maximum and when queens have a maximal egg-laying rate^33,40,41^. We thus hypothesised that, when protein is in high demand by queens and larvae—such as during the execution period—workers would eliminate supernumerary queens to enhance larval development. Interestingly, we found that protein deprivation did not provoke queen executions in the Argentine ant, which suggests that factors unrelated to diet are in operation.

With regards to queen fate, our results are consistent with those of a recent study^36^, which suggested that Argentine ant workers cull queens based on their CHC profiles. We found that surviving queens had different CHC profiles than executed queens, and it appears that two di-methyl alkanes were the main compounds responsible for these differences. Compared to executed queens, surviving queens bore significantly greater amounts of 5,11-diMeC_29_ and 5,11-diMeC_33_. Interestingly, Abril *et al*.^36^ also observed large amounts of these two di-methyl alkanes in the CHC profiles of queens sampled at the end of the execution period (i.e., surviving queens). This result indicates that, in the Argentine ant, it is likely that a queen’s CHC profile determines her fate and that workers use the di-methyl alkanes 5,11-diMeC_29_ and 5,11-diMeC_33_ as signals in the culling process. Greater amounts of di-methyl alkane 5,11-diMeC_29_ are associated with higher egg-laying rates and ovarian indices in Argentine ant queens^36^, which suggests that workers could use this CHC to selectively kill the least productive queens, as occurs in *Lasius niger*^42^. This latter species founds colonies via pleometrosis, and levels of the methylalkane 3MeC_31_ and of the alkene C_31:1_, compounds that are both correlated with queen productivity and maturity, were found to be higher in surviving queens than in executed queens^42^. Later, it was discovered that 3MeC_31_ was both a primer pheromone (i.e., affecting worker reproduction) and a releaser pheromone (i.e., affecting worker behavior, notably by increasing aggressiveness towards queens)^43^. Consequently, the di-methyl alkanes identified here could serve as releaser queen pheromones in the Argentine ant (i.e., queen pheromones that affect the behaviour of other nestmates) and thus increase worker aggressiveness towards queens.

In the Argentine ant, queens experiencing the same set of experimental conditions, whether monogynous or polygynous, displayed marked variation in their egg-laying ability^15^. Furthermore, when experimental conditions were polygynous as opposed to monogynous, the oviposition rates of queens shifted: there were always one or two main queens who laid the eggs, and the contribution of other queens was small to null^15,30^. These results underscore that there might be some form of reproductive inhibition among queens. This hypothesis is supported by the fact that Argentine ant queens can regulate colony social organization using queen pheromones^26,44,45^. It is thus possible that, in this species, reproductive competition occurs among a colony’s queens and leads to the execution of the least productive individuals during the queen execution period.

Interestingly, Abril *et al*.^36^ found greater amounts of di-methyl alkanes 5,11-diMeC_29_ and 5,11-diMeC_33_ in young queens than in the executed queens reported in the present study (i.e. old queens). This finding suggests that the elimination of old queens to allow gyne production in the Argentine ant could serve as a mechanism to increase queen productivity in the colony. A previous study on the fecundity of Argentine ants found that the egg-laying of young queens was similar to that of the old ones^46^. However, young queens in this study underwent all of their maturation in the laboratory and this may have influenced the process of fat accumulation, and, therefore, their fecundity. As the CHC profile reflects the hormonal state more than the current egg-laying rate^47^, the results obtained in the present study provide new information about the biological reasons beyond the annual execution period in the Argentine ant.

Further research is necessary to assess whether the compounds mentioned above are, in fact, queen pheromones that help regulate queen numbers and, ultimately, whether they have a role in suppressing egg laying activity by fellow queens in polygynous situations. It is also important to identify the inhibitory queen pheromone present in this highly polygynous ant species. This information will allow us to gain a broader understanding of the social organization of polygynous ant species and inform strategies for managing Argentine ant invasions, potentially through the use of pheromones that induce colony self-destruction.

## Methods

### Effect of temperature and queen number on queen executions

We collected 156 queens from 36 different Argentine ant nests before the execution period (in early March and April) along the southern edge of the Gavarres Massif, near the village of Castell d’Aro (NE Iberian Peninsula) (41°49′N3°00′E). In this area, there are invaded and uninvaded cork oak secondary forests. In the invaded forests, Argentine ant colonies can cover up to 323 ha^48^ and mainly occur under stones. Here, we use Heller *et al*.’s^49^ definition of an Argentine ant colony: a group of interacting nests. From October to June, Argentine ant nests within a radius of 3–4 m aggregate in large clusters. Therefore, the nests sampled were at least 10 meters apart to ensure that they truly represented different colonies. In the laboratory, we created 12 monogynous colonies: each contained one queen and approximately 300 workers. We also created 24 polygynous colonies: 12 contained four queens, and 12 contained eight queens. Each polygynous colony had the same ratio of workers to queens—approximately 300 workers per queen. In these artificial colonies, the queen(s) and workers were from the same nest of origin. The colonies were housed in plaster structures (180 × 115 × 25 mm high) and were fed the Bhatkar diet^50^ and mealworms on a daily basis. To prevent the ants from escaping, the inner sides of the structure were coated with liquid PTFE (Fluon). The colonies were kept under conditions of 80% relative humidity.

To test the effect of temperature and queen number on queen executions, four monogynous colonies, four polygynous colonies with four queens, and four polygynous colonies with eight queens were placed in environmental chambers kept at one of three temperatures (±SD): 24 °C ± 0.1, 28 °C ± 0.1, and 32 °C ± 0.1. We chose these temperatures because we observed in previous studies that workers executed queens with varying frequency within this range. We checked the colonies daily for a period of three weeks to count the number of executed queens.

### Pattern of queen executions in the field

We characterised queen execution patterns in the field, by comparing queen numbers during May 2013 between an area along the invasion front where the Argentine ant was still in contact with the native ant community (hereafter, contact zone) and an area almost exclusively occupied by the Argentine ant (hereafter, invaded zone). These two areas were located in cork oak secondary forests in the NE Iberian Peninsula; the first was at the southern edge of the Gavarres Massif, near Santa Cristina d’Aro (41°48′51.71″N; 3°01′50.57″E), and the second was in the Cadiretes Massif, near Pedralta (41°47′31.53″N; 2°58′52.79″E). Colony queen numbers are known to differ between these two areas: they were found to be higher in the contact zone than in the invaded zone^30^.

In the Argentine ant, new colonies are founded via fission in mid-February or early March^51–53^. Consequently, the drop in queen numbers reported during the month of May could be caused by queen executions, as reported by Keller *et al*.^20^, since no other ecological or biological processes have been observed during this period that could decrease queen numbers^51–53^. For this reason, we assumed in this study that the decline in queen numbers in May was the result of queen executions.

To compare queen numbers during the execution period in these two areas, we collected 2-litre soil samples from a total of 24 nests in the contact zone and 24 nests in the invaded zone. To ensure sample independence, the nests sampled were at least 10 m apart. Samples were taken weekly during the whole month of May (six nests were sampled in each area every week). We assumed that the execution period had ended when queen numbers remained stable for two consecutive weeks, which had occurred by the last week of May. All the queens present in these samples were counted *in situ* in the field by manual extraction of the queens present in each sample.

The contact zone and the invaded zone were ±1 km apart and had similar environmental characteristics, which meant that differences in queen numbers were unlikely to be related to abiotic differences between the areas.

### Effect of protein deprivation on queen executions

To investigate the effect of protein deprivation on queen executions, we collected 12 nests before the execution period (in early April) from the same location as for the temperature/queen number experiment. We created 12 experimental colonies, each containing 15 queens and approximately 100 workers per queen. They also all contained larvae from several developmental stages (mainly from earlier stages so that larvae require more food from workers). Specifically, 0.1 g of larvae were weighed to the nearest 0.01 mg using a digital analytical balance. They were then carefully introduced into the experimental colonies. In these artificial colonies, the queen(s), workers and larvae were from the same nest of origin. Each experimental colony was housed in a rectangular plastic box (17 × 11 × 3 mm high) with a water source (a plastic tube filled with water that was wicked through a cotton ball). To prevent the ants from escaping, the inner sides of the box were coated with liquid PTFE (Fluon). The colonies were kept at 26 °C ± 0.1 and under conditions of 80% relative humidity; they were exposed to approximately 1 h of light per day (i.e., the time needed for feeding and cleaning). The twelve colonies were fed on a daily basis, but their diet differed: six received the Bhatkar diet, a sugar solution, and *ad libitum* mealworms, and six received only the sugar solution. We checked the nests daily for three weeks to count the number of executed queens.

### Relationship between CHC profiles and queen fate

We extracted the CHCs of the queens from the artificial colonies used in the temperature/queen number experiment. First, we collected 19 executed queens that had remained in the colony for 0–24 hours until their corpses were found. Second, we collected 19 queens that had survived the three weeks of observations. As a control, we also sampled 24 queens before the execution period (in early April) from the same location as for the temperature/queen number experiment. All the samples were frozen (−20 °C) until CHC extraction could occur. The queens’ gasters were eliminated to prevent any contamination by the Dufour gland. The samples were thus composed of just their heads, thoraces, and legs. We immersed the corpses in 50 μl of dichloromethane (GC grade) for 10 min. They were then removed, and the resulting extract was stored at −20 °C until further analysis. The extracts were evaporated and then redissolved in 50 μl of dichloromethane containing eicosane (8 ng/μl) as an internal standard. Next, 1 μl of each extract was injected into a gas chromatography (GC; Agilent 7820 A Series, Agilent Technologies, USA) equipped with a HP-5 capillary column (30 m × 0.32 mm × 0.25 µm) and a flame ionization detector. Sample analysis was performed using helium as a carrier gas; the flow rate was set to 2 ml/min (34.9 cm/sec); an injection volume of 1 µl with a split ratio of 1:5 was used; and the inlet temperature was set to 275 °C. The following temperature program was used: 75 °C held for 0 min; an increase from 75 °C to 200 °C at a rate of 20 °C min-1; then another increase from 200 °C to 315 °C at a rate of 5 °C min-1; and 315 °C held for 15 min. The FID detector was set to a temperature of 300 °C. CHCs were identified using standard alkanes, compound libraries, and Kovats retention indices. CHC quantities were determined using the eicosane area as the internal standard (ng per head and thorax [i.e., one queen]).

Because we were analysing the CHCs of executed queens that may have remained in their nests for as long as 24 hours before being collected, we conducted an experiment to determine if queen CHC profiles degraded over time. We collected 6 nests containing 24 queens from the same location as in the temperature/queen number experiment. We created artificial colonies that each contained 1 queen and 300 workers; they were kept under the same conditions as the colonies in the temperature/queen number experiment. We randomly assigned 12 queens to the experimental group and 12 queens to the control group. In the experimental group, queens were freeze killed, warmed back up to ambient temperature, and returned to their artificial colonies of origin for 24 hours. Their corpses were then frozen again until the CHC analysis could be conducted. In the control group, queens were left unharmed in their artificial colonies for 24 hours. They were then freeze killed, thawed, and frozen again until the CHC analysis could be conducted. We found no significant differences between the CHC profiles of experimental queens and those of control queens (R = 0.43, Wilks’ λ = 0.815 χ^2^_14_ = 2.10, p = 0.73) (Fig. 4). Furthermore, the two groups did not differ in their total quantities of CHCs (GLMM t_22_ = −1.54, p = 0.19) or the quantities of the two main CHCs responsible for the differences in the CHC profile between surviving and executed queens (5,11-diMeC_29_: GLMM: t = 0.27, p = 0.79; 5,11-diMeC_33_: GLMM: t = −0.54, p = 0.61). These results indicate that the CHC profiles of Argentine ant queens did not significantly degrade even if dead queens remained in the nest for 24 hours prior to being collected.

### Statistical analyses

Generalised linear mixed models (GLMMs) were performed to compare the percentage of executed queens (binomial error distribution and logit link function) based on the ambient temperature, the number of queens present, and protein availability (presence/absence). We included colony as a random factor. When overall significant differences were detected, pairwise comparisons were performed using t-tests employing pooled standard deviation.

The numbers of queens in the contact zone and the invaded zone were analysed using GLMMs (Poisson error distribution and log-link function). The response variable was queen number. The area (contact zone or invaded zone) and time (specific week of May) were fixed factors, and location (Santa Cristina d’Aro or Pedralta) was a random factor. We used the function glmmPQL in the MASS package to carry out the GLMMs.

To examine the queens’ CHC profiles, we obtained the relative contribution of each CHC peak to the total. Only identified peaks whose mean relative quantities were above 2% in at least one of the groups of queens were used. Discriminant analyses were then performed to investigate how CHC profiles differed between executed and surviving queens. To identify the CHCs that characterised each group, principal component analysis (PCA) was used. We focused on the CHCs demonstrating the greatest differences—those whose factor loadings on the first axis had an absolute value > 0.70. We further investigated these data using GLMMs in which the response variable was the mass of a given peak in nanograms (calculated by comparing the area of the peak with that of the internal standard) and colony was a random factor. To ensure normality, all the variables were log-transformed prior to being used in the discriminant analyses and the PCA. In addition, Levene’s test was used to determine whether the variables displayed homogeneity of variance; only those that did were retained. We used the function lme in the nlme package to carry out the GLMMs.

All the statistical analyses were performed using R^54,55^.

## Data Availability

The datasets generated and analyzed during the current study are available from the corresponding author on reasonable request.
